# Mast cells co-expressing CD68 and inorganic polyphosphate are linked with colorectal cancer

**DOI:** 10.1371/journal.pone.0193089

**Published:** 2018-03-15

**Authors:** Stella Arelaki, Athanasios Arampatzioglou, Konstantinos Kambas, Efthimios Sivridis, Alexandra Giatromanolaki, Konstantinos Ritis

**Affiliations:** 1 Laboratory of Molecular Hematology, Democritus University of Thrace, Alexandroupolis, Greece; 2 Department of Pathology, University General Hospital of Alexandroupolis, Alexandroupolis, Greece; Universita degli Studi di Bari Aldo Moro, ITALY

## Abstract

Inflammation is a hallmark of colorectal cancer (CRC). Neutrophils are well-known mediators in tumor biology but their role in solid tumors, including CRC, was redefined by neutrophil extracellular traps (NETs). Given that it was recently demonstrated that platelet-derived polyP primes neutrophils to release NETs, we examined surgical specimens from CRC to investigate the presence of polyP, as a possible NET inducer. Biopsies with adenomas, hyperplastic polyps, inflammatory bowel disease and healthy colon tissues were used as controls. In all cases, the presence of polyP was apparent, with the main source of polyP being the mast cells. In all CRC and all adenomas with high-grade dysplasia, a substantial number of mast cells, more than 50%, co-expressed intracellularly polyP with CD68 surface antigen (CD68+), but this was not the case in the other examined disorders. PolyP-expressing mast cells were detected in close proximity with tumor cells and neutrophils, suggesting polyP expression by CD68+ mast cells among the stimuli which prime neutrophils to release NETs, in CRC. Moreover, the detection of CD68+ polyP-expressing mast cells could represent a potential prognostic marker in colorectal adenomas and/or carcinomas.

## Introduction

Inflammation is a hallmark of solid tumors, including colorectal cancer (CRC)[1,2]. It is possible that the recruitment and/or activation of inflammatory cells, such as lymphocytes, macrophages and mast cells, is defined by the tumor milieu, although the way by which these cells interact with cancer cells remains ambiguous[3]. Neutrophils in particular were an understudied cell population in cancer and their implication in pathological processes was restricted mainly to infection and inflammation[4–6].

The role of neutrophils in tumor pathology was redefined by neutrophil extracellular traps (NETs), a mechanism that was initially described as pure antimicrobial[7]. To date, there is increasing evidence that NET deposition in the tumor mass is associated with tumor progression[8–10]. Moreover, it has been suggested that NETs might favor metastasis[11,12] and they have also been implicated in cancer-associated thrombosis[9,13].

Recently, in our laboratory, we demonstrated that inorganic polyphosphate (polyP), a linear polymer composed of numerous high-energy phospho-anhydride-bonded orthophosphate units, activates neutrophils to release NETs in thromboinflammation[14]. PolyP has been implicated in a number of physiological and non-physiological processes, namely in proliferation[15], apoptosis[16], energy metabolism[17], inflammation[18,19], angiogenesis[20], blood clotting[19,21], cancer-associated thrombosis[22] and tumor metastasis[20,23] and it is expressed by neoplastic cells[22,24], bacteria[21], platelets[25] and mast cells[18].

In this study, we provide evidence that polyP exists in CRC and adenomas with high-grade dysplasia, expressed mainly by CD68-positive (CD68+) mast cells and located in close proximity with neutrophils. We also suggest that polyP staining, combined with immunostaining for CD68, is tumor specific and could be possibly used as a prognostic marker in CRC and adenomas.

## Materials and methods

### Human samples

The material comprised of formalin-fixed paraffin-embedded surgical tissue specimens from 10 patients with CRC and as control samples there were used biopsy materials from 6 adenomas with high- and 6 with low-grade dysplasia, 6 hyperplastic polyps, 6 diagnosed with ulcerative colitis, 6 with Crohn’s disease, and 6 from normal colonic tissue. Patient characteristics are demonstrated in **S1 Table**.

Blocks were retrieved from the surgical pathology files of the Department of Pathology, Democritus University of Thrace, Medical School, Alexandroupolis, Greece. The tissues had been fixed in 10% formaline and processed conventionally to paraffin wax. Written consent was granted by all individuals involved in this study. The study protocol design was in accordance with the Declaration of Helsinki and was approved by the Ethics Review Board of the University Hospital of Alexandroupolis.

### Fluorescence and immunofluorescence staining

Tissue sections were cut at 3 μm and were dewaxed and rehydrated through graded alcohols. For heat-induced epitope retrieval the sections were placed in citrate buffer (1:10 dilution, pH 7.2) and heated in microwaves for 10 minutes. After washes in phosphate buffer saline (PBS) (2 x 5 minutes) the non-specific sites of the sections were blocked with 5% goat serum (Invitrogen, Carlsbad, USA; PCN5000; 1/200 dilution) in 2% BSA-PBS. The slides were then incubated overnight at 4°C with different primary antibodies (for further details see **S2 Table)**. After washes in PBS (2 x 5 minutes), the sections were incubated with secondary antibodies for 45 minutes in room temperature. A goat anti-rabbit Alexa fluor 647 antibody (Invitrogen, Carlsbad, USA; A-21244; 1/500 dilution), a rabbit anti-mouse Alexa fluor 647 antibody (Invitrogen, Carlsbad, USA; A-21239; 1/500 dilution) or a rabbit anti-mouse Alexa fluor 488 antibody (Invitrogen, Carlsbad, USA; A-11059; 1/500 dilution) were utilized as secondary antibodies. The slides were subsequently washed in PBS for 2 x 5 minutes and counterstained with Propidium Iodide (Sigma-Aldrich, St. Louis, USA; 556463; 1/1000 dilution) for 5 minutes. After a thorough washing in PBS, the sections were incubated with JC-D8 fluorescence probe in 40 mM HEPES buffer[26] for polyP detection, as previously described[14].

Since the emission wavelength of JC-D8 probe is approximately at 525 nm and nuclear staining by DAPI or Hoechst interferes with probe reactivity[26], thus eliminating any use of 405 laser, we were unable to perform a double immunostaining in combination with staining for polyP and nuclei.

In immunofluorescence staining with two primary antibodies, no JC-D8 probe was used. Sections were mounted and directly visualized in a confocal microscopy (Spinning Disk Andor Revolution Confocal System, Ireland) in a PLAPON 606O/TIRFM-SP, NA 1.45 and UPLSAPO 100XO, NA 1.4 objectives (Olympus, Hamburg, Germany).

In each section, the CD68+ or the tryptase-positive (tryptase+) cells were enumerated and quantified, from a total of 50 polyP-expressing cells. The scores were analyzed as demonstrated in the statistical analysis section.

All the cases were examined by at least two independent observers, who were blinded to the patient, histopathological and outcome data. The results were not related to tumor size, tumor grade, number of lymph nodes involved with metastasis, and patients’ survival.

### Statistical analysis

Statistical analyses were performed using one-way analysis of variance (ANOVA) with Scheffé test for post hoc comparisons, following a test for normal distribution of sample populations (Sapiro-Wilk). P values less than 0.05 were considered significant. All statistical analyses were performed with OriginPro 8.

### Hybrid staining protocol

Tissue sections were cut at 3 μm, deparaffinized and placed in low pH antigen retrieval target solution (DAKO, Agilent, Santa Clara, USA; K8005; pH 6) followed by microwaving for 3 x 5 minutes. After washes in phosphate buffer saline (PBS) (2 x 5 minutes), the non-specific sites of the sections were blocked with 5% goat serum in 2% BSA-PBS for 45 minutes and the slides were then incubated for 60 minutes with the primary antibody CD68 (DAKO, Agilent, Santa Clara, USA; M0814). Slides were then washed with PBS (2 x 5 minutes) and endogenous peroxidase activity was neutralized using the EnVision Flex Peroxidase Block (DAKO, Agilent, Santa Clara, USA; SM801) for 10 minutes. Following washes in PBS (2 x 5 minutes), the sections were incubated with the EnVision FLEX+ Mouse (LINKER; DAKO, Agilent, Santa Clara, USA; K8021) for 15 minutes to amplify the signal of the primary mouse antibody. Slides were subsequently washed in PBS and the secondary antibody EnVision Flex/HRP (DAKO, Agilent, Santa Clara, USA; SM802) was applied for 30 minutes. After washes in PBS (2 x 5 minutes), the color reaction was developed in 3,3’-diaminobenzidine (DAB) for 6 minutes. After a thorough wash in PBS the sections were incubated with JC-D8 fluorescence probe for polyP in 40 mM and visualized by standard light/fluorescence microscopy.

## Results

### PolyP is present in human colon cancer

Since it has been recently indicated that NETs are abundant in sections of human CRC[10] and considering that polyP, a naturally occurring molecule, was recently identified as a NET inducer[14], we investigated whether polyP is present in human colon adenocarcinoma.

**Fig 1:**
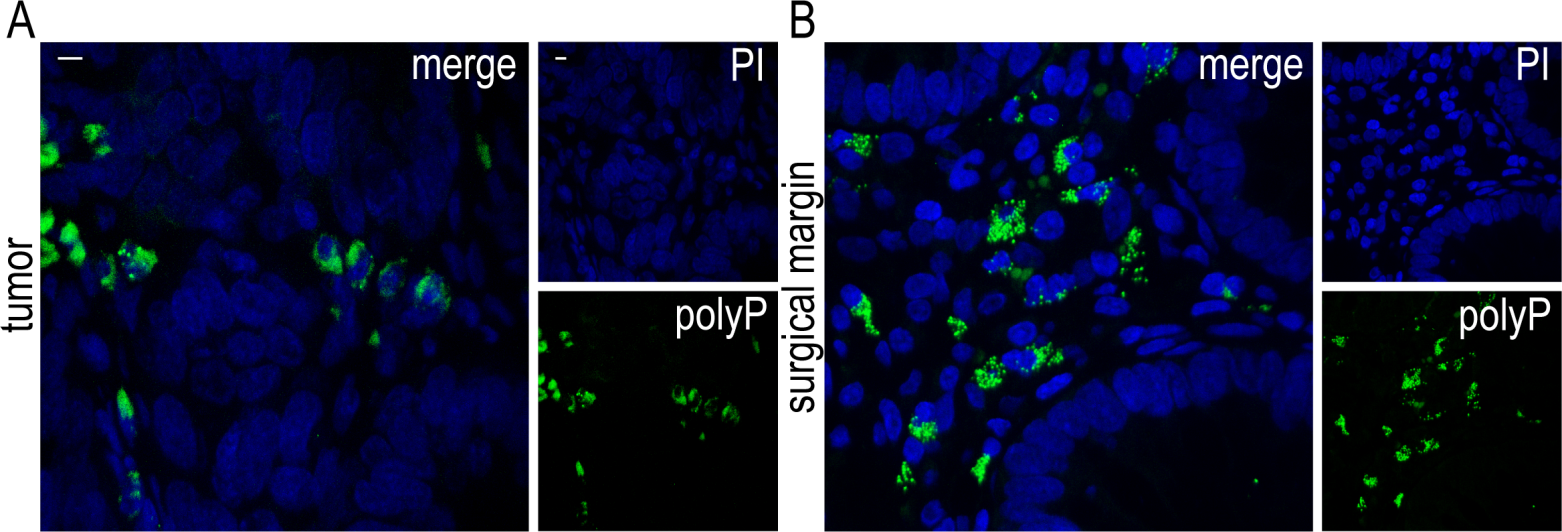
Presence of polyP in human colon cancer. Confocal microscopy for polyP staining in sections of colonic adenocarcinoma specimens from (A) the main mass and (B) the surgical margin. Blue: PI, Green: JC-D8 polyP-specific fluorescent probe. One representative out of ten independent experiments is shown. Original magnification x600, Scale bar– 5μm.

We examined sections of colectomy specimens from ten patients with adenocarcinoma, obtained from both the main mass and the surgical margin. All examined sections demonstrated strong cytoplasmic staining for polyP in large mononuclear cells, with the pattern of polyP localization being in cytoplasmic granular structures **(Fig 1A and 1B)**. The polyP-expressing cells were large cells with a large eccentric nucleus and abundant cytoplasm, thus morphologically different from platelets.

In tumor mass sections, the polyP-transferring cells were detected in the stroma, surrounding the cancer cells and infiltrating excessively all the layers of the bowel, while in surgical margin sections the same cells were noted in the mucosa next to glandular epithelial cells **(Fig 1A and 1B)**.

The aforementioned mononuclear cells were observed as the main source of polyP in CRC.

### Mast cells are identified to express polyP in colonic adenocarcinoma

Based on the above findings, we sought to identify the polyP-expressing cells. Since they were mononuclear cells present in tumor microenvironment, mainly in stroma, we conducted immunofluorescence staining using markers for mast cells[18] (mast cell tryptase), macrophages (CD68), plasma cells[24] (CD38), T- and B- lymphocytes (CD3, CD20 and CD79a) and neutrophils (NE). Moreover, we examined CK20, a specific marker for colon cancer cells, α-SMA for stroma myofibroblasts, and CD61 for platelets[25].

**Fig 2:**
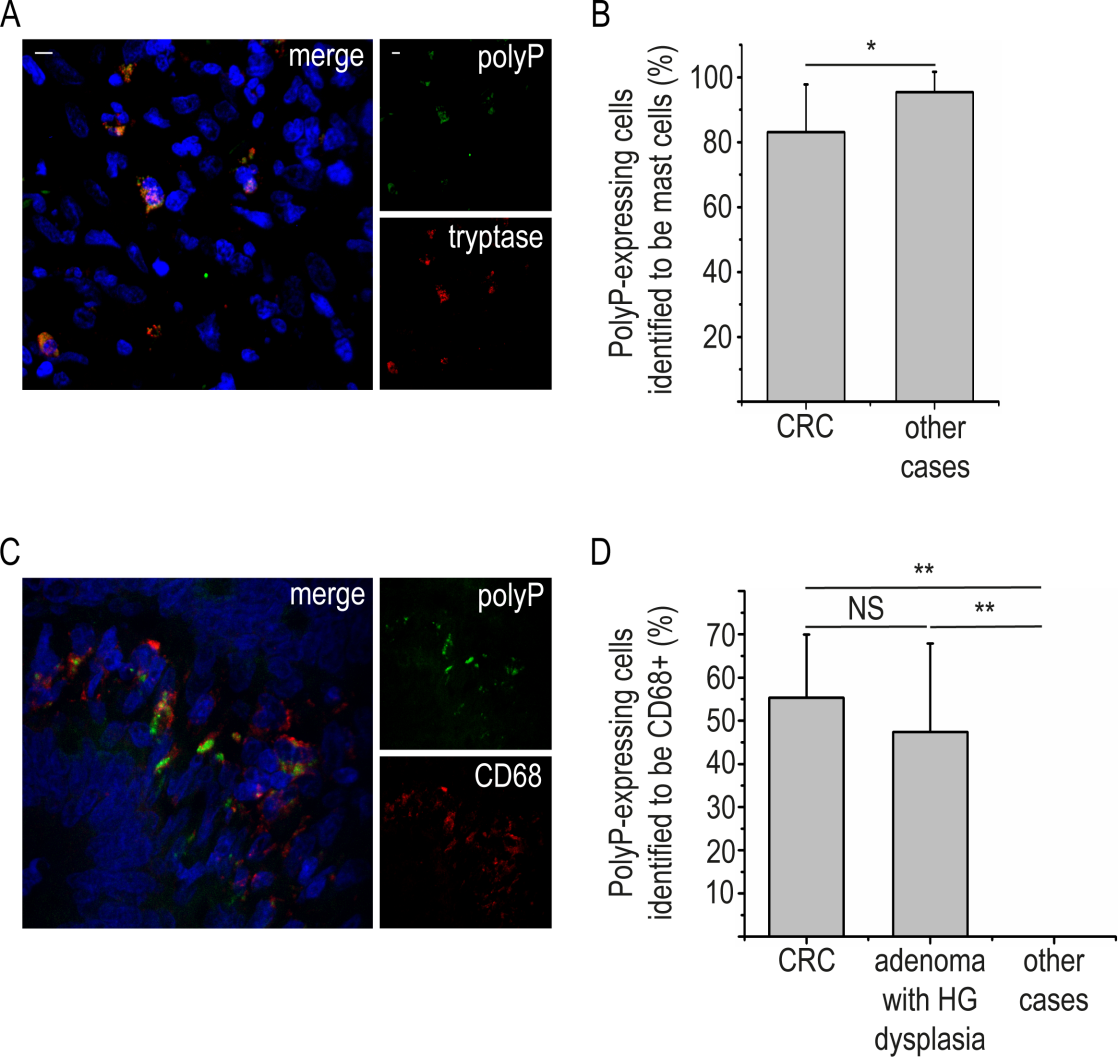
PolyP-expressing cells are positive for mast cell tryptase and CD68 in human colon cancer. Confocal microscopy for **(A)** polyP / tryptase and **(C)** polyP / CD68 staining in sections of colonic adenocarcinoma specimens. **(B and D)** Data from six independent experiments presented as mean ± SD. Blue: PI, Green: (A and C) JC-D8 polyP-specific fluorescent probe, Red: (A) mast cell tryptase or (C) CD68. (A and C) One representative out of ten independent experiments is shown. Original magnification x600. Scale bar– 5μm. *P < 0.05, **P < 0.001, NS–not significant, HG–high grade.

**Fig 3:**
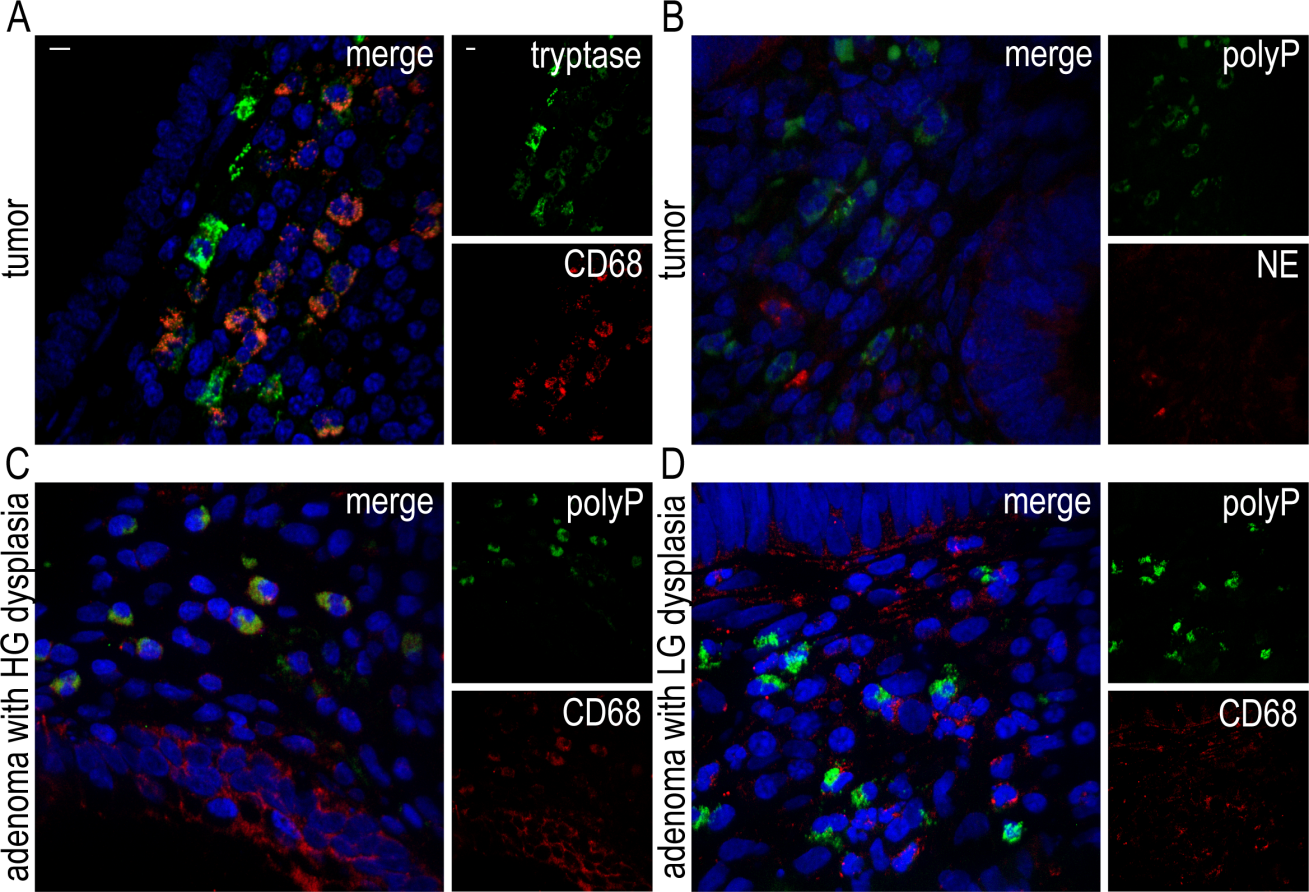
CD68+ mast cells are important sources of polyP in CRC and adenomas with high-grade dysplasia, and are localized next to neutrophils in CRC. Confocal microscopy for **(A)** Tryptase / CD68, **(B)** polyP / NE and **(C and D)** polyP / CD68 staining in sections of **(A and B)** colonic adenocarcinoma specimens and adenomas with **(C)** high- or **(D)** low-grade dysplasia. Blue: PI, Green: (A) mast cell tryptase or (B and D) JC-D8 polyP-specific fluorescent probe, Red: (A, C and D) CD68 or (B) NE. One representative out of (A and B) ten or (C and D) six independent experiments is shown. Original magnification x600. Scale bar– 5μm. HG–high grade, LG–low grade.

In each examined section, 83% (± 15%) of polyP-expressing cells were positive for mast cell tryptase **(Fig 2A and 2B, Table 1)** and 55% of polyP-positive cells (± 15%) demonstrated reactivity with CD68 **(Fig 2C and 2D, Table 1)**. All polyP-expressing cells were negative for the other examined markers. Double immunofluorescence staining for CD68 and tryptase in combination with staining for polyP and nuclei was not feasible, due to technical limitations, as previously reported in the materials and methods section. Thus, we used a double immunostaining protocol to examine the co-expression of CD68 and tryptase, given that almost all polyP-expressing cells are mast cells. Co-localization of CD68 and tryptase was observed in 58% (± 13%) of mast cells **(Fig 3A)**, which was in accordance with the above-mentioned percentage of CD68+ polyP-expressing cells (55 ± 15%). Moreover, staining with NE indicated that polyP-expressing cells were in close proximity with neutrophils and their remnants **(Fig 3B).**

**Table 1:** Identification of the polyP-expressing cells in CRC, in pre-malignant and in non-malignant conditions of the colon.

	*Specimen studied*							
*Markers tested for co-expression*	TM	SM	HGA	LGA	HP	UC	CD	N
**PolyP / Mast cell tryptase**	+	+	+	+	+	+	+	+
**PolyP / CD68**	+	+	+	-	-	-	-	-
**Mast cell tryptase / CD68**	+	+	+	+*	+*	+*	+*	+*

**TM**: tumor mass; **SM**: surgical margin; **HGA**: adenoma with high-grade dysplasia; **LGA**: adenoma with low-grade dysplasia; **HP**: hyperplastic polyp; **UC**: ulcerative colitis colonic tissue; **CD**: crohn’s disease colonic tissue; **N**: normal colonic tissue; **+**: presence of cells co-expressing the tested markers; **-**: absence of cells co-expressing the tested markers. +*: There was a debate regarding the occasional presence of cells co-expressing the tested markers in these tissues.

Together, these data indicate that among mast cells, the stained with CD68 surface marker constitute the main cellular source of polyP, in CRC, and are localized in tumor stroma next to cancer cells and neutrophils.

### CD68+ polyP-expressing cells are detected in pre-malignant colon but not in non-malignant conditions

The above findings led us to investigate the co-expressions of CD68 as possible biomarkers in CRC. Double immunostaining for CD68 and mast cell tryptase was examined first. Since occasional co-localization was observed in the non-malignant disorders and there was discrepancy among the independent observers, we sought to clarify whether the detection of cytoplasmic expression of polyP by CD68+ cells in colon could prove to be more reliable. In all cases examined, polyP-expressing cells were positive for mast cell tryptase **(Fig 2B, Table 1)**. However, CD68+ polyP-expressing cells were detected only in CRC and adenomas with high-grade dysplasia **(Fig 3C)**, while in the adenomas with low-grade dysplasia **(Fig 3D)**, and in non-neoplastic cases, polyP-expressing cells were consistently CD68-negative **(Fig 2D, Table 1)**. Therefore, the detection of CD68+ polyP-expressing cells rather than CD68+ mast cells could be used as a potential prognostic tool for colorectal adenoma or adenocarcinoma.

Alternatively, we used the same markers, CD68 and polyP, in an effort to simplify and accelerate the detection procedure. We tested and consequently optimized a staining method that enables the evaluation of a biopsy by using a standard fluorescence microscope. Thus, tumor mass and healthy colon sections were stained using a single hybrid protocol which combines immunohistochemistry for CD68 and fluorescence staining for polyP. In accordance with our previous results, CD68+ polyP-expressing cells were identified only in CRC sections **(S1A and S1B Fig)**.

## Discussion

This is the first time that polyP was shown to be expressed by CD68+ mast cells in malignant and pre-malignant environment, namely that of colonic adenocarcinoma and adenomas with high-grade dysplasia.

In line with previous studies demonstrating that colon adenocarcinoma is a malignancy tightly correlated with inflammation, our finding that polyP is expressed by mast cells in colonic tumor microenvironment suggests that polyP possibly participates in the crosstalk between inflammatory and cancer cells[27–32]. To date, most studies have focused on the prohemostatic, prothrombotic and proinflammatory role of polyP, demonstrating a highly anionic polymer that is secreted by activated platelets and triggers both thrombosis and inflammation. Our recent study demonstrated that polyP secreted by thrombin-activated platelets induces NET release. In another study of ours, NET structures were detected in human colon adenocarcinoma sections[10]. These previous data along with our findings that mast cells are main sources of polyP in CRC and they are in close proximity with neutrophils, imply a possible interplay between CD68+ polyP-expressing mast cells and neutrophils. Thus, we suggest that CD68+ polyP-expressing mast cells could crosstalk with neutrophils through polyP, leading to NET generation and their subsequent implication in tumor biology.

Although the biological significance of polyP in the clinical outcome of human malignancies remains obscure, the identification of CD68+ polyP-expressing cells only in CRC sections and adenomas with high-grade dysplasia, but not in the non-malignant conditions of the colon, which was unexpected, could indicate the possibility of a novel prognostic biomarker. Considering that some adenomatous polyps do not undergo malignant transformation, the fact that in our study only a few of the examined adenomatous polyps, those with high-grade dysplasia, exhibited CD68+ polyP-expressing cells might be related to precancerous behavior of these polyps. Herein, it should be mentioned that the independent observers had different estimation regarding the occasional detection of CD68+ mast cells in non-neoplastic disorders. As a result, the detection of CD68+ polyP-expressing cells exclusively in neoplastic conditions of colorectal tissue could be promising for assessment as a prognostic tool in CRC and adenomas.

In conclusion, our data indicate that the exclusive presence of CD68+ polyP-expressing cells in colon adenocarcinoma and adenoma sections is a specific marker of the neoplastic colon and could possibly provide a potential prognostic marker. However, large series of CRC and adenoma cases should be studied, in order to elucidate if the detection of CD68+ polyP-expressing cells could contribute to the prognostic significance for the clinical outcome of CRC and whether their presence in adenomas is related to high risk for colorectal carcinoma development. Moreover, our study suggests a possible crosstalk between mast cells and neutrophils in the tumor microenvironment, through polyP, leading possibly to the previously reported NET release.

## Supporting information

S1 Fig A hybrid staining method for the detection of CD68+ polyP-expressing cells. A combination of CD68 immunohistochemical and polyP fluorescence staining in sections of **(A)** CRC and **(B)** normal mucosa. Red circle: CD68 and polyP co-localization, Black rectangle: staining with CD68, Green triangle: staining with JC-D8 polyP-specific fluorescent probe. One representative out of two independent experiments is shown. Original magnification x400.
(TIF)

S1 Table Clinical characteristics of the patients1,2 that participated in the study. (DOCX)

S2 Table List of the primary antibodies used in the study. (DOCX)
