# Supplementary figures and images for: Mast cells co-expressing CD68 and inorganic polyphosphate are linked with colorectal cancer
Source: PLoS One. 2018 Mar 15;13(3):e0193089. doi: 10.1371/journal.pone.0193089 (PMC5854234; doi:10.1371/journal.pone.0193089)

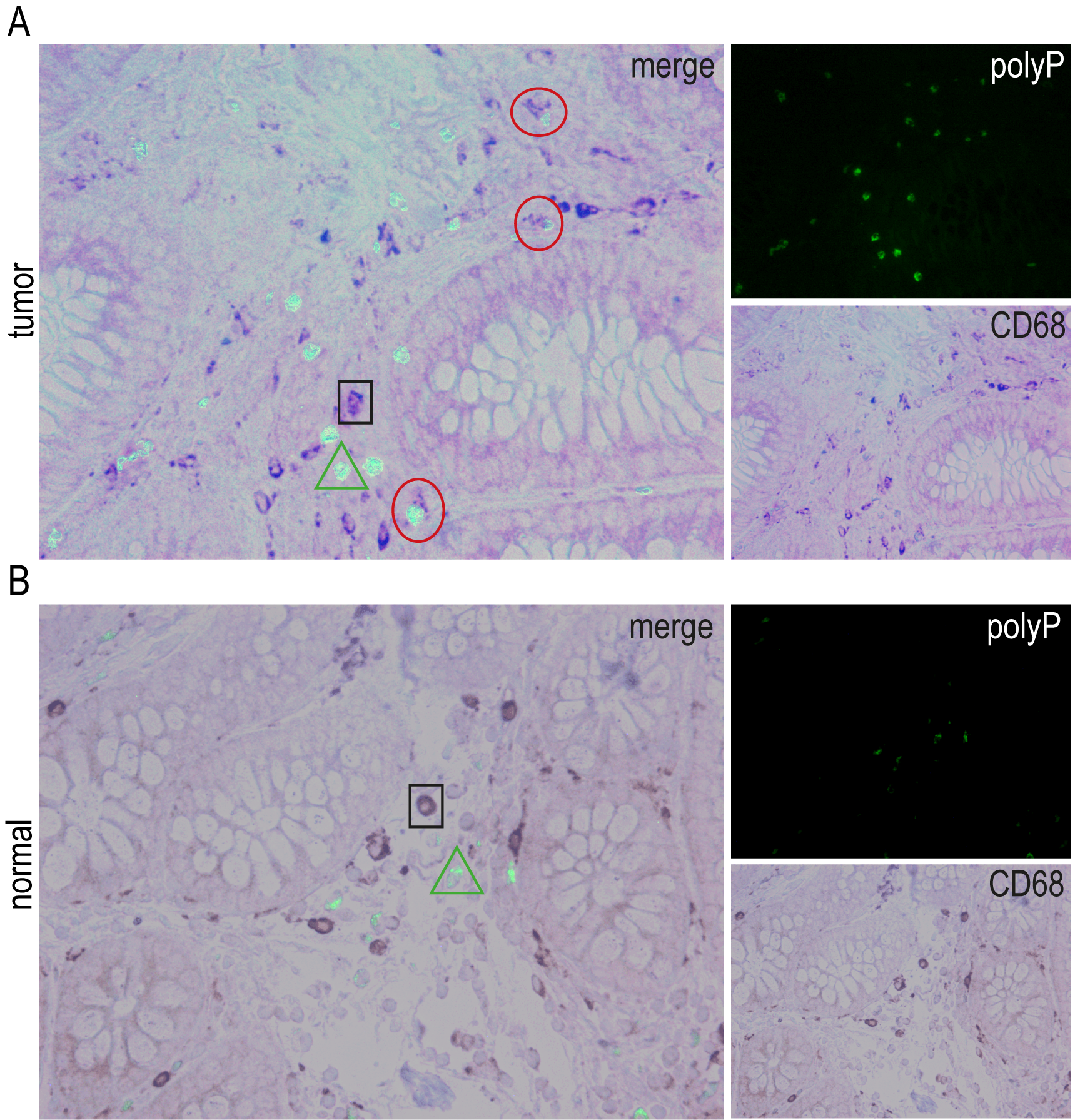

Supplement: S1 Fig — A combination of CD68 immunohistochemical and polyP fluorescence staining in sections of (A) CRC and (B) normal mucosa. Red circle: CD68 and polyP co-localization, Black rectangle: staining with CD68, Green triangle: staining with JC-D8 polyP-specific fluorescent probe. One representative out of two independent experiments is shown. Original magnification x400. (TIF) [file pone.0193089.s001.tif]
