# Supplementary material for: Mast cells co-expressing CD68 and inorganic polyphosphate are linked with colorectal cancer
Source: PLoS One. 2018 Mar 15;13(3):e0193089. doi: 10.1371/journal.pone.0193089 (PMC5854234; doi:10.1371/journal.pone.0193089)
Supplement: S1 Table — (DOCX) [file pone.0193089.s002.docx]

**S1 Table:** Clinical characteristics of the patients^1,2^ that participated in the study.

| **Case** | **Number of patients**  **examined** | **Patient age in years**  **(mean ± SD)** | **Patient gender  (number of females / males)** |
| --- | --- | --- | --- |
| Colorectal cancer^3^ | 10 | 73.6 ± 7.8 | 3 / 7 |
| Adenoma with  high-grade dysplasia | 6 | 67.5 ± 14.4 | 2 / 4 |
| Adenoma with  low-grade dysplasia | 6 | 68.5 ± 7.8 | 1 / 5 |
| Hyperplastic polyp | 6 | 64.7 ± 11.6 | 2/ 4 |
| Ulcerative colitis | 6 | 33.5 ± 11.1 | 3 / 3 |
| Crohn’s disease | 6 | 39.7 ± 18.3 | 0 / 6 |
| Normal mucosa | 6 | 42.8 ± 22.8 | 2 / 4 |
| **Total** | 46 | 57.3 ± 20.2 | 13 / 33 |

^1^ All the samples were randomly selected.

^2^ No patient received chemotherapy, or radiotherapy, or was suffering from another inflammatory, autoimmune or infectious disease.

^3^ In patients with CRC, there was no correlation between our results and the tumor staging.
