# Supplementary material for: Mast cells co-expressing CD68 and inorganic polyphosphate are linked with colorectal cancer
Source: PLoS One. 2018 Mar 15;13(3):e0193089. doi: 10.1371/journal.pone.0193089 (PMC5854234; doi:10.1371/journal.pone.0193089)
Supplement: S2 Table — (DOCX) [file pone.0193089.s003.docx]

**S2 Table:** List of the primary antibodies used in the study.

| **Primary**  **antibody** | **Clonality** | **Provider** | **Catalogue**  **number** | **Clonality/Host** | **Dilution** |
| --- | --- | --- | --- | --- | --- |
| Mast cell tryptase | AA1 | DAKO, Agilent,  Santa Clara, USA | M7052 | Monoclonal/Mouse | 1:1000 |
| CD68 | - | Abcam.  Cambridge, UK | Ab125212 | Polyclonal/Rabbit | 1:200 |
| CD68 | KP1 | DAKO, Agilent,  Santa Clara, USA | M0814 | Monoclonal/Mouse | 1:100 |
| CK20 | Ks20.8 | DAKO, Agilent,  Santa Clara, USA | M7019 | Monoclonal/Mouse | 1:150 |
| CD61 | Y2/51 | DAKO, Agilent,  Santa Clara, USA | M0753 | Monoclonal/Mouse | 1:100 |
| CD3 | F7.2.38 | DAKO, Agilent,  Santa Clara, USA | M7254 | Monoclonal/Mouse | 1:100 |
| CD20 | L26 | DAKO, Agilent,  Santa Clara, USA | M0755 | Monoclonal/Mouse | 1:200 |
| CD79a | JCB117 | DAKO, Agilent,  Santa Clara, USA | M7050 | Monoclonal/Mouse | 1:200 |
| CD38 | SPC32 | Leica BIOSYSTEMS,  Wetzlar, Germany | NCL-L-CD38-290 | Monoclonal/Mouse | 1:100 |
| a-SMA | 1A4 | DAKO, Agilent,  Santa Clara, USA | M0851 | Monoclonal/Mouse | 1:100 |
| NE | - | Santa Cruz,  CA, USA | sc-25621 | Polyclonal/Rabbit | 1:200 |
